# Odor-active aroma compounds in traditional fermented dairy products: The case of mabisi in supporting food and nutrition security in Zambia

**DOI:** 10.1016/j.crfs.2025.100976

**Published:** 2025-01-16

**Authors:** Thelma W. Sikombe, Anita R. Linnemann, Himoonga B. Moonga, Stefanie Quilitz, Sijmen E. Schoustra, Eddy J. Smid, Anna Alekseeva

**Affiliations:** a Food Microbiology, Wageningen University and Research, P.O. Box 17, 6700 AA, Wageningen, the Netherlands; b Food Quality and Design, Wageningen University and Research, P.O. Box 17, 6700 AA, Wageningen, the Netherlands; c Laboratory of Genetics, Wageningen University and Research, P.O. Box 17, 6700 AA, Wageningen, the Netherlands; d Department of Food Science & Nutrition, School of Agricultural Sciences, University of Zambia, P.O. Box 32379, Lusaka, Zambia

**Keywords:** Traditional fermentation, Odor-active compounds, GC-O-MS, Microbial community, Amplicon sequencing

## Abstract

Aroma is a key sensory attribute that determines consumer preference and acceptability of foods. The aroma of fermented dairy products comprises the volatile organic compounds (VOCs) produced by the activity of fermenting microbes and the compounds originally present in unfermented raw milk. A unique combination of specific compounds detectable by human olfactory senses creates the distinct odor profile of fermented products. This study investigated the influence of different production methods on the VOCs responsible for the odor-active compounds, and the microbial communities present in mabisi, a traditional Zambian fermented dairy product. The VOCs and microbial community composition of four mabisi variants were investigated using GC-O-MS and PTR-QiTOF-MS techniques, and 16S rRNA amplicon sequencing, respectively. A panel of three assessors identified the odor-active compounds from the GC-O-MS, and the compound's quantitative aspects were obtained by the PTR-QiTOF-MS.

Twelve volatile compounds were identified as odor-active compounds during the GC-O-MS analysis. The most prominent were ketones and esters, which imparted a buttery and fruity aroma, respectively. The PTR-QiTOF-MS run identified and quantified a total of 390 m/z peaks, 55 of which were tentatively identified. 16S rRNA amplicon sequencing revealed a diverse microbial community, with *Lactococcus* species dominating. While the VOC profiles showed significant variation in functionality among the variants, minor differences were observed in microbial composition.

The study confirms that high compound concentration does not necessarily correlate with compound odor activity. Our findings offer insights into the significance of aromas and microbial ecology to support optimization strategies for upscaling traditional fermented products.

## Highlights

• Bovine milk was the substrate used to produce mabisi, a traditional Zambian food.
• Volatile organic compounds were studied in relation to different processing methods.
• GC-O-MS and PTR-QiTOF-MS were applied as complementary research techniques.
• Aroma profiles varied across variants while the microbial composition did not vary.
• 16S rRNA amplicon sequencing revealed dominance of *Lactococcus* species.

## Graphical abstract

**Figure:**
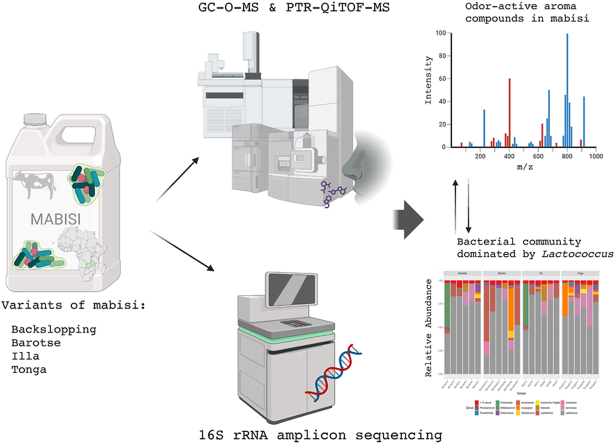

## 1 Introduction

Aroma is an essential food attribute, especially in traditionally fermented varieties, where it adds distinctive characteristics valued by consumers. Its importance spans sensory appeal, cultural significance, quality and safety indication, product differentiation, health considerations, and formulation optimization (Sousa et al., 2022; Sikombe et al., 2023; Boscaini et al., 2003). Traditional fermentation is integral to the food culture of many low-income countries worldwide, where numerous foods rely on spontaneous fermentation with an undefined mix of microbes. Understanding the link between the microbial community and the produced aromas is key to successfully promoting these foods. However, such fermented foods have been understudied due to the traditional and informal nature of their production (Tamang et al., 2020; Obafemi et al., 2022). Our study investigated this relationship using mabisi, a spontaneously fermented bovine milk product from Zambia as an archetypal example. Mabisi's unique flavor comes from a mix of volatile and non-volatile organic compounds (VOCs), primarily generated by the activity of lactic acid bacteria (LAB) (Schoustra et al., 2013).

Recent studies on mabisi have outlined its diverse VOC composition, highlighting the presence of organic acids, alcohols, esters, and carbonyl compounds (Moonga et al., 2021). LAB, particularly *Lactobacillus* and *Lactococcus*, are the dominant species in mabisi (Schoustra et al., 2013). Similar findings are reported in other African milk products like amasi, nunu, and masai, albeit with regional variations (Akabanda et al., 2013; Isono et al., 1994; Osvik et al., 2013). VOC formation results from the enzymatic activities of coexisting microorganisms that degrade the milk components (Dan et al., 2017; Smid et al., 2014). The nature and concentration of VOCs determine the distinct flavors that influence consumer perception and acceptability (Clark, 1998). Only a fraction of the volatiles, however, significantly contributes to the aroma that is perceived by human olfaction (Blank and Marsili, 1996; Baldovini et al., 2020; Song et al., 2018).

Previous research used Headspace Solid-Phase Microextraction Gas Chromatography-Mass Spectrometry (HS-SPME-GC-MS) to profile the volatile compounds of different variants of mabisi. In this study, we employed Gas Chromatography-Olfactometry-Mass Spectrometry (GC-O-MS) to identify odor-active aroma compounds and this was complemented with real-time VOC analysis using Proton Transfer Reaction-Quadrupole interface Time-of-Flight Mass Spectrometry (PTR-QiTOF-MS) to gain insights on compound concentrations (Yener et al., 2016). The study examined four variants of traditional mabisi: backslopping, barotse, illa, and tonga mabisi, which differed in their methods of production. The variations in the production methods included termination of fermentation process after curd formation (tonga variant), use of an aliquot from the previous fermentation as inoculum to initiate the next fermentation cycle (backslopping variant), agitation of the fermentation system coupled with the removal of the butter that forms on top (illa variant), and the alternate removal of whey and addition of fresh milk during the fermentation process (barotse variant) (Moonga et al., 2019). The study aimed to identify specific volatile compounds that influence sensory perception through olfactometry and characterize bacterial communities using 16S rRNA amplicon sequencing. We also explored the correlation between the volatile compounds and the microbial community to understand their relationship and establish volatile compound development in mabisi.

The findings of our study are crucial for identifying the VOCs that distinguish these traditional products and highlight how unique fermentation practices contribute to the aroma complexity. This knowledge is valuable to guide future efforts to optimize flavor and enhance the quality and consistency of these traditionally fermented products, as well as others, for ultimate upscaling.

## 2 Materials and methods

### 2.1 Mabisi samples preparation

**Fig. 1:**
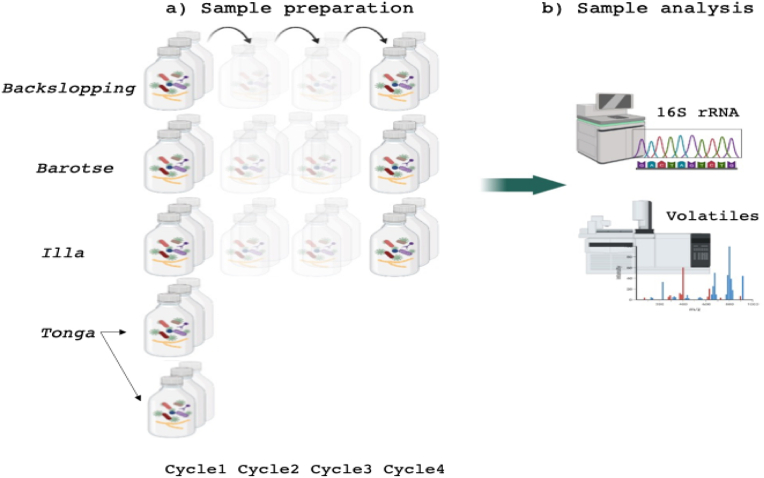
Experimental design for the four mabisi variants. (a) Sample preparation: Backslopping, barotse, and illa were produced by ‘backslopping’ the first ripe product three more times as indicated by the “shadowed” bottles whereas tonga was prepared by two separate batches. Analysis samples were taken from the first and fourth-cycle products, and the two separate batches in the case of tonga. (b) Sample analysis: Collected samples were subjected to bacterial profiling with 16S rRNA amplicon sequencing and VOC analysis with GC-O-MS and PTR-QiTOF-MS.

Four variants of mabisi namely, backslopping, barotse, illa, and tonga, were examined. Samples from the first and fourth production cycles referred to as backslopping1 & 4, barotse1 & 4, and illa 1 & 4, were collected for the three variants. Tonga was an exception as it does not follow production cycles; instead, its samples were collected from two separate production days and were referred to as tonga 1 & 2. For the three variants backslopping, barotse and illa, the first cycle comprised of first ripe products. For the backslopping and illa variants, samples were collected after inoculation with an aliquot from the previous successful product, while barotse samples were collected after whey removal and raw milk addition. The fourth cycles were obtained after repeating these procedures three more times (Fig. 1a). Samples were prepared in the kitchen at the University of Zambia's Department of Food Science and Nutrition, following previously outlined methods (Sikombe et al., 2023; Moonga et al., 2019). Subsequently, samples were frozen (−20 °C), shipped to Wageningen University (Netherlands), and stored at −80 °C until further analysis (Fig. 1b). Analytical measurements were conducted in triplicate.

#### 2.1.1 GC-O-MS analysis

Two GC ovens, linked by a transfer line at 220 °C, were employed alongside the TSQ9000 Triple Quadrupole MS (Thermo Scientific™, Waltham, USA). Detection was facilitated by the MS and a PHASER Pro GC-Olfactory Port (GL Sciences, Eindhoven, the Netherlands), both equipped with a transfer line at 220 °C. Additionally, a cryo cold trap (Cryotherm, Kirchen, Germany) was integrated into the system. Compounds were desorbed from the fiber for 2 min and separated on a polar column (Stabilwax®-DA, 30 m length, 0.25 mm ID, 0.5 μm df Restek, Bellefonte, USA). A Programmable Temperature Vaporizing (PTV) inlet, heated to 250 °C, served as the sample inlet and operated in split mode at a 50:50 ratio for simultaneous acquisition of odor characteristics by panelists at the sniffing port and compound identification in MS mode. The GC oven temperature began at 35 °C for 5 min, raised to 240 °C at a rate of 10 °C/min, and maintained for 5 min. Helium was the carrier gas at a constant flow rate of 1.2 mL/min. Mass spectral data was collected over a mass-to-charge ratio (m/z) range of 33–250 in full-scan mode at 3.0030 scans/sec.

Mass spectral data were analyzed using Chromoleon® 7.2.10 (Thermo Scientific™, Waltham, USA). The NIST 2014 main library was used for component matching. An alkane standard solution (Sigma-Aldrich, Darmstadt, Germany) with pentane and heptane was measured with an SPME fiber and direct injection, and the retention times were used for calculating the Linear Retention Index (LRI) using the following formula:

$$LRI=100.\left[z+\left(\frac{tri-trz}{tr\left(z+1\right)-trz}\right)\right]\text{,}$$

where tr is the retention time; z is the previous alkane number; z+1 is the following alkane number and ‘i’ is the target compound.

The GC-O effluent was sniffed by a panel of three non-smoking female assessors, aged 29.7 ± 4.5 years. Each assessor evaluated two samples per day, with at least a 2.5 h break between samples to prevent olfactory fatigue. To minimize odor interference, assessors refrained from wearing perfume and avoided strongly scented foods, such as garlic, the evening before and on the day of testing. The compounds were identified with MS and GC-O responses were recorded using synchronized audio tape during sample injection. The detected odors were described and recorded by the assessors. The odor descriptors were matched with peaks based on compound retention and assessor response times. The responses were considered valid when at least two assessors could detect the odors at a given time. The odors perceived by only one assessor were considered noise. Compound identification and odor descriptions were verified using the LRI and the Flavornet databse (Acree and Arn, 2004).

#### 2.1.2 PTR-QiTOF-MS analysis

Two ml samples in 250 ml beakers were agitated in a water bath at 25 °C for 30 min. Samples were measured in a PTR-QiTOF-MS (Ionicon Analytik gmbh, Innsbrück, Austria) for 60 s, with an acquisition rate of one spectrum/sec (m/z range 0.00–570). Measurements were initiated with a 5 s flushing of the PTR machine with ambient air as a blank, followed by sample measurement. The measurement was conducted in Vmode in the following ionization conditions: ion source voltage Us and Uso of 145.0 and 76.6 V, respectively, drift voltage of 999.0 V, drift temperature of 60 °C and drift pressure of 3.79 mbar corresponding to an E/N value of 134 Townsend. A mass resolution above 4000 was held throughout the run. An internal reference standard with peaks at m/z 203.943 and 330.856 was continuously injected using the PerMaScal device.

The data were blank-corrected by t-tests, removing masses with no significant difference to the blank and those with a significant negative difference. This reduced m/z peaks from 534 to 390.

#### 2.1.3 Microbial composition

Mabisi DNA extraction followed Schoustra et al. (2013) protocol. DNA quality and concentration were assessed using NanoDrop™ ND-2000 and Qubit™ 4 fluorometer (Thermo Scientific, UK). Samples were sent to Novogene (UK) for V3-V4 hypervariable region 16S rRNA amplicon sequencing on Illumina NovoSeq 6000. PCR amplification used primers 341F and 806R, and amplicons were pooled, end-repaired, A-tailed, and ligated with Illumina adaptors. Paired-end reads were trimmed and filtered with fastp, FLASH, and DADA2. Chimeric sequences were removed with Vsearch. QIIME2 generated Amplicon Sequenced Variants (ASVs) using the Silva database. Phylogenetic relationships were examined with QIIME2 by aligning multiple sequences. ASV abundance was rarefied to a standard read number, and the abundance table was obtained at kingdom to genus levels.

### 2.2 Statistical analysis

Means and standard deviations for all data sets were calculated in Excel. All other analyses were performed in R Version 4.3.1. The VOC data were median normalized and ANOVA was performed, followed by a post-hoc Tukey test. Principal component analysis (PCA) and Heatmaps to visualize the VOC data were performed using the FactoMineR package (Version 2.9) and pheatmap package (Version 1.0.12) respectively. The Phyloseq package (Version 1.46) was used for microbiome data analysis, while a correlation between the VOCs and the bacterial taxa was performed using the Spearman correlation coefficient. Significance for all analyses was considered at *P* < 0.05.

## 3 Results

### 3.1 Volatile compounds detected by the GC-O-MS

The GC-O-MS results show variations in compound diversity and peak area percentages, indicating differences in VOC types and quantities (Table S1). Twenty-six VOCs were identified, including esters, ketones, hydrocarbons, aldehydes, alcohols, carboxylic acids, hydrocarbons, and sulphur-derived compounds. While significant differences were found between the variants for most VOCs except for acetone, diacetyl, limonene, and 2-nonanone (P < 0.05), differences between product cycles were only detected in barotse samples.

**Fig. 2:**
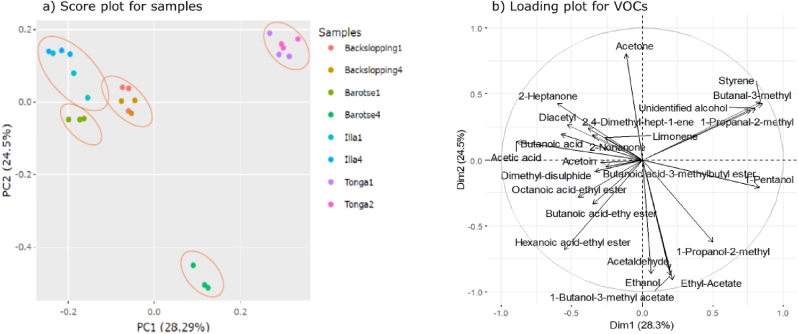
PCA plot of the peak area percentages of volatiles identified by GC-O-MS in four variants of mabisi and specific compounds related to each variant. (a) Score plots showing the clusters of the mabisi variants with each sample represented by a dot. (b) Loading plots of the projection of different VOC compounds.

The first two components of the PCA of VOC data explained 52.8% of the total variation, revealing five distinct clusters (Fig. 2). While samples from different production cycles clustered together across the variants, barotse1 formed a separate cluster from barotse4, with the latter projecting onto the negative dimension of PC2. Barotse1, illa, and backslopping samples projected on the negative dimension of PC1 while tonga samples were separated from other variants along the positive dimension of PC1.

The most dominant compounds were alcohols, particularly, ethanol and 1-pentanol. Barotse4 correlated highly with 1-butanol-3-methyl acetate, ethanol, ethyl acetate, 2-methyl-1-propanol, and acetaldehyde, while barotse1 and the illa samples were closely related to acetic acid, butanoic acid, and 2-nonanone. Ethanol, acetic acid, 2-nonanone, butanoic acid, octanoic acid, acetaldehyde, and ethyl acetate distinguished the samples from the two production cycles of barotse, that is barotse 1 and 4. Tonga samples were associated with 2-methyl-1-propanal, 3-methyl-butanal, styrene, and an unidentified alcohol. Acetoin, dimethyl disulfide, and ethyl butanoic acid were distinctive compounds for backslopping samples.

#### 3.1.1 Odor-active volatile compounds detected by olfactometry

**Fig. 3:**
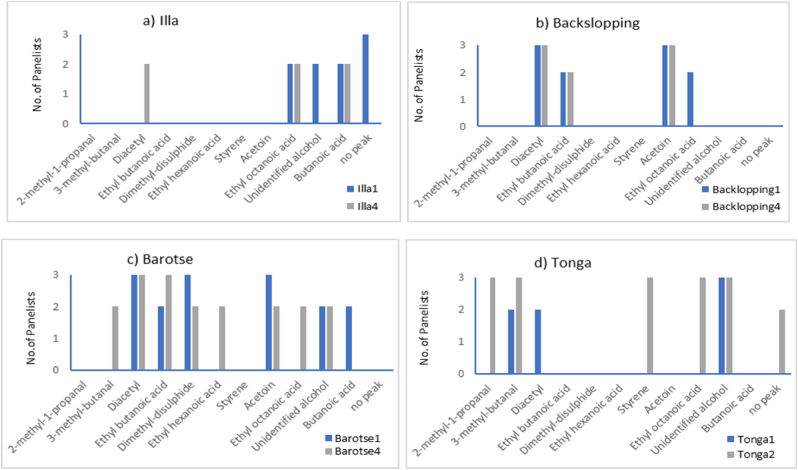
Sniffing bar charts of volatile compounds. (a) Illa, (b) Backslopping, (c) Barotse, and (d) Tonga obtained by gas chromatography−olfactometry (GC-O) using a panel of three assessors. The x-axis represents the identified volatile compounds and the y-axis represents the number of assessors that could identify a particular compound.

A total of 12 volatile compounds were detected by olfactometry and their odor profiles were described by a panel of assessors. Of the twelve compounds, one could not be detected by the GC-MS. Only the compounds that were detected by at least two of the panelists were considered odor-active and contributors to the characteristic aroma of mabisi. The identified odor-active volatile compounds and the number of panelists that could detect an odor at the sniffing port of the GC-O at a given time are presented in Fig. 3. The compounds included 2-methyl-1-propanal, 3-methyl-butanal, diacetyl, ethyl-butanoic acid, dimethyl-disulphide, ethyl-hexanoic acid, styrene, acetoin, ethyl octanoic acid, butanoic acid, and unidentified alcohol. Each variant of mabisi, however, had its own distinct set of compounds comprising the odor-active profile. Barotse4 had the highest number of perceivable odors with eight odor-active volatiles, while barotse1 and tonga2 each had six compounds identified. Similarly, backslopping1 and illa1 contained four identified odor-active compounds each. Diacetyl and ethyl octanoic acid were the most common odor-active volatiles as they were detected in all four variants of mabisi.

The odor-active compounds identified by olfactometry and the linear retention index on the Stabilwax®-DA column with their odors as described by the panelists are further presented in Table 1. Diacetyl was characterized by sour, buttery, caramel, creamy, and fruity notes, while ethyl octanoic acid imparted fruity and sour aroma notes to mabisi. Like diacetyl, acetoin was described by a typical buttery caramel, creamy, sweet, and sour aroma. Interestingly, despite acetoin being detected in the MS mode in all the variants except tonga, its odor activity was only perceived in barotse and backslopping samples. Likewise, ethyl butanoic acid was detected in the MS mode in all four mabisi variants but could only be perceived by olfactory senses in barotse and backslopping samples. Ethyl butanoic acid, like the other esters, contributed sweet, fruity, and fatty aroma notes to mabisi.

**Table 1:** Key odor-active compounds of four variants of mabisi, including their LRI as calculated on the stabilwax column, and odor descriptors by the GC-O-MS the panelists.

Volatile compound	Mean RT	Mean LRI^a^	Odor description by panelists^c^
Propanal, 2-methyl	7572	823,4	unpleasant, sharp, pungent^a^, hazelnut
Butanal, 3-methyl	9097	932,16	creamy, cocoa^a,c^, nutty^a^
Diacetyl	10,078	993,65	buttery^a,b,c,d^, caramel, creamy^b,c^, sour, sweet^e^, burnt milk
Butanoic acid, ethyl ester	10,98	1053,55	fruity^b,c^, ripe lemon, sweet^c^, strawberry, apple^a^
1-Propanol, 2-methyl	11,733	1103,07	strongly fruity^a^, grape, sulphury
Dimethyl disulphide	11,752	1104,82	sharp, swampy, floral, vinegar
Hexanoic acid, ethyl ester	14,032	1248,8	banana^b,c^, weakly fruity^a,b,c^, sour
Styrene	14,616	1288,31	soapy, floral, buckwheat, herbal, mushroom
Acetoin	14,981	1313,94	buttery^a,c^, creamy^a^, sour, caramel
Octanoic acid, ethyl ester	16,83	1450,82	sharp, fruity^a,c^, sour
Unidentified alcohol	17,55	1505,55	cooked meat, unpleasant, stuffy, mushroom, rancid, putrid, sour, pungent, slightly sulphury, creamy, salty caramel, toasted almonds
No peak^b^	n.a	n.a	sharp, cinnamon, rancid, old smoke
Butanoic acid	19,293	1645,01	rancid^a,b^, cheesy^a,b^, stale

a LRI, linear retention index of the compounds on a Stabilwax® DA column.

b No peak was detected by the GC-MS, but the panelists perceived and described an odor through olfactometry.

c References: ^a^ (Acree and Arn, 2004); ^b^ (Friedrich et al., 1998); ^c^ (Mariaca et al., 2001); ^d^ (Ott et al., 1997); ^e^ (Boscaini et al., 2003).

Butanoic acid was the only carboxylic acid with odor activity in mabisi, imparting rancid, cheese, stale, and weakly cardboardy aroma perceived in barotse1 and illa4. Two branched-chain aldehydes, 3-methyl-butanal and 2-methyl-1-propanal, were also identified as odor-active volatiles in barotse and tonga samples. These aldehydes contributed an overall nutty flavor, despite apparent differences in their characteristic aromas. To illustrate, while a creamy aroma was attributed to 3-methyl-butanal, 2-methyl-1-propanal was characterized by a pungent and somewhat unpleasant aroma.

Certain compounds were exclusively found in some mabisi variants but not others. For instance, ethyl-hexanoic acid was only perceived in barotse4 while dimethyl-disulphide, described as having a "sharp, swampy, and floral" aroma, was present in both cycles of the barotse samples. Similarly, styrene, characterized by a soapy, floral, buckwheat, herbal, and mushroom-like aroma, was unique to tonga2 samples. Additionally, an unidentified alcohol contributed to a range of aromas in both barotse and tonga samples, including cooked meat, sweaty, rancid, putrid, sour, pungent, sharp, slightly sulphury, salt caramel, and toasted almonds. Tonga2 and illa1 had an odor referred to as ‘no peak’ as it was perceived by the panelists and not detected in MS mode. The ‘no peak’ was described as having flower, sharp, cinnamon, rancid, and old smoke aroma.

### 3.2 Volatile compounds detected by the PTR-QiTOF-MS

**Fig. 4:**
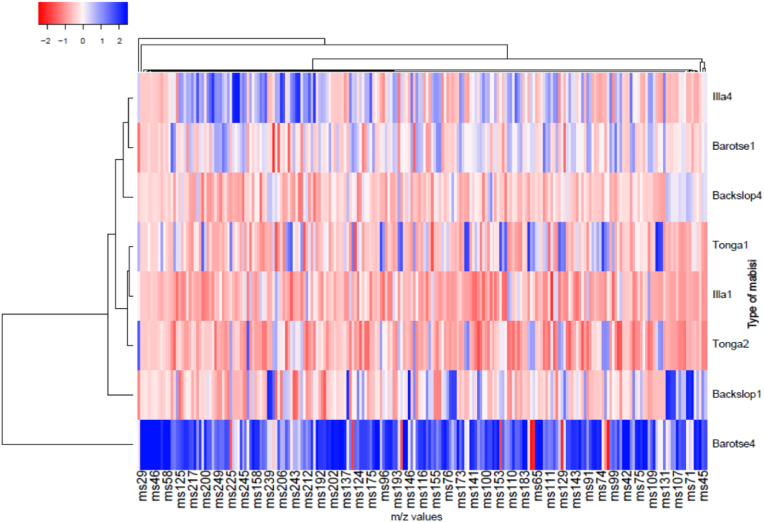
Heatmap matrix of the normalized VOC data for the four variants of mabisi obtained by PTR-QiTOF-MS. The x-axis represents mass-charge-ratio (m/z) and the y-axis represents the mabisi variants. An intense blue color signifies a high concentration, while an intense red color signifies a low concentration.

We used the PTR-QiTOF-MS to quantify headspace compounds and complement the GC-O-MS findings to gain insights into VOC's quantitative aspects. As expected, the PTR-QiTOF-MS results revealed a more complex volatile profile than the GC-O-MS. A heatmap depicting the intensity (i.e., normalized concentrations in ppbv) for each of the mass peaks (Fig. 4), and cluster analysis (Fig. S2) demonstrated the ability of PTR-QiTOF-MS to detect the variability between the cycles of barotse, backslopping, and illa. While backslopping1 had higher concentrations and a more diverse range of volatiles than backslopping4, the fourth cycles for illa and barotse exhibited greater diversity and concentrations compared to their respective first cycles. Low volatile concentrations were observed in all tonga samples. Barotse4 had the highest volatile concentrations and variations. Peaks that significantly differentiated the samples and those unique to specific samples were identified (Table S2). Consistent with heatmap results, the VOC concentrations in backslopping decreased over cycles, this could be further investigated to optimize the quality of backslopping mabisi.

#### 3.2.1 Tentative identification of m/z peaks to complement the GC-O-MS analysis

Fifty-five m/z were tentatively identified by comparing PTR-QiTOF-MS masses with the NIST library and literature (Table S2). A mass deviation of 0.001 was allowed as a threshold. Since multiple compounds can have the same or similar mass, a peak may correspond to more than one compound at the same time (Yener et al., 2016; Soukoulis et al., 2010; Bottiroli et al., 2020; Aprea et al., 2015).

The mass peak at m/z 89.0602 significantly distinguished the backslopping samples and was tentatively identified as acetoin, ethyl acetate, or butanoic acid due to their similar similar molecular weights. However, acetoin particularly stood out when comparing the PTR-QiTOF-MS and the GC-O-MS results. It was also perceived as an odor-active compound in backslopping by olfactometry. Therefore, the peak at m/z 89.0602 is most likely acetoin. Additionally, the mass peak at m/z 87.0440, identified as diacetyl, was significantly higher in backslopping1 and exhibited odor activity in all mabisi variants.

The compounds with mass peaks at m/z 45.0336 and m/z 47.0492 were tentatively identified as acetaldehyde and ethanol. These compounds had the highest peaks during the PTR-QiTOF-MS run, and their presence was most prominent in barotse4. While acetaldehyde did not have the highest peak during GC-O-MS analysis, ethanol in barotse4 was still the highest peak in the GC-O-MS analysis.

Other peaks, among them, m/z 145.1232 and m/z 117.0911 tentatively identified as ethyl hexanoic acid (which could also be octanoic acid) and ethyl butanoic acid (which could be hexanoic acid) significantly differentiated barotse4 from the other mabisi products. These compounds were also identified as odor-active volatiles. In the illa samples, the mass peak at m/z 117.0911, tentatively identified as butanoic acid ethyl, was distinctive for illa4. A compound with a mass peak at m/z 87.0801, tentatively identified as butanal-3-methyl, was distinctive for tonga1. However, this peak could also represent 2-pentanone or pentanal. However, since butanal-3-methyl was distinctive for tonga and an odor-active volatile for both tonga samples, it is likely that this particular mass represents butanal-3-methyl.

Only two mass peaks had concentrations above 1 ppm, specifically m/z 47.0492 (ethanol) and m/z 45.0336 (acetaldehyde), both of which were associated with barotse4. The other peaks, including m/z 72.0516, 87.044, 87.0801, 94.0727, 105.0708, 117.0911, 145.1232, and 173.1521 (propanal-2-methyl, diacetyl, butanal-3-methyl, dimethyl-disulphide, styrene, ethyl butanoic acid, ethyl hexanoic acid, and ethyl octanoic acid, respectively), had concentrations below 10 ppbv. The mass peak at m/z 89.0602, tentatively identified as acetoin/butanoic acid, had higher concentrations ranging between 7.3 and 151 ppbv across the different mabisi variants.

### 3.3 Taxonomic identification of the bacterial communities in mabisi

**Fig. 5:**
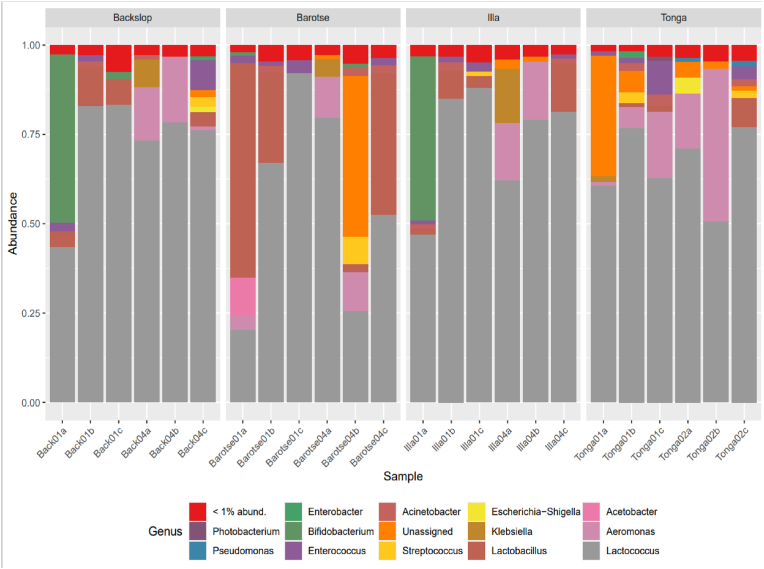
Taxonomic identification of the bacterial communities in the four mabisi products showing the relative abundance at the genus level. The different colors represent the most abundant genera of the taxonomic units.

The 16S rRNA amplicon sequencing results showed a diverse community of bacteria dominated by the phylum *Pseudomonadota* and *Bacillota.* The bacteria were taxonomically classified up to the genus level. Bacterial taxa were visualized using bar plots and a total of 14 genera were identified, while the species with an abundance below 1% were assigned to others (Fig. 5).

In general, species of the genera *Lactococcus*, *Aeromonas, Acetobacter, Lactobacillus, Klebsiella, Escherichia-shigella, Streptococcus, Acinetobacter,* and *Enterococcus*, in order of relative abundance, made the top ten species in the samples. Among the bacterial genera identified, *Lactococcus* was dominant in all the samples with relative abundances ranging from 20 to 90%, stressing the importance of this species as an acid producer in milk fermentation. After *Lactococcus*, members of the genus *Lactobacillus* dominated backslopping1, barotse1 and 4, and illa1 whereas *Aeromonas* was the most abundant genus in backslopping4. *Aeromonas* also dominated the tonga and illa4 samples. Some species of *Bifidobacterium* were observed in barotse and illa samples.

**Fig. 6:**
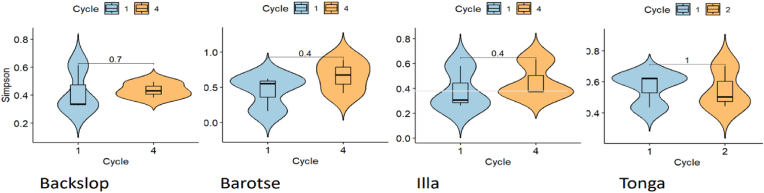
Simpson alpha diversity - indices showing variation in the bacterial diversity between cycles of the mabisi variants. p > 0.05.

Alpha diversity analysis, as determined by the Simpson index, revealed that barotse4 and tonga1 exhibited the highest microbial diversity, whereas backslopping1 and illa1 displayed the lowest diversity (Fig. 6). In general, the microbial diversity for the first cycles across all the products except for tonga mabisi was lower than their subsequent cycles. The variation in microbial diversity between cycles was slightly more pronounced in the barotse compared to the other variants. However, no significant difference in diversity was evident between the respective cycles of the variants (p > 0.05). Regarding the Observed alpha diversity, illa1, and tonga1 exhibited a greater microbial community richness (Fig. S2). Interestingly, only barotse samples displayed increased species richness in subsequent cycles, with the “Observed” alpha diversity. The species richness of the other variants was most evident in the first-cycle samples. Similarly, no significant differences were observed across the cycles of the mabisi variants in terms of “Observed” alpha diversity (p > 0.05).

**Fig. 7:**
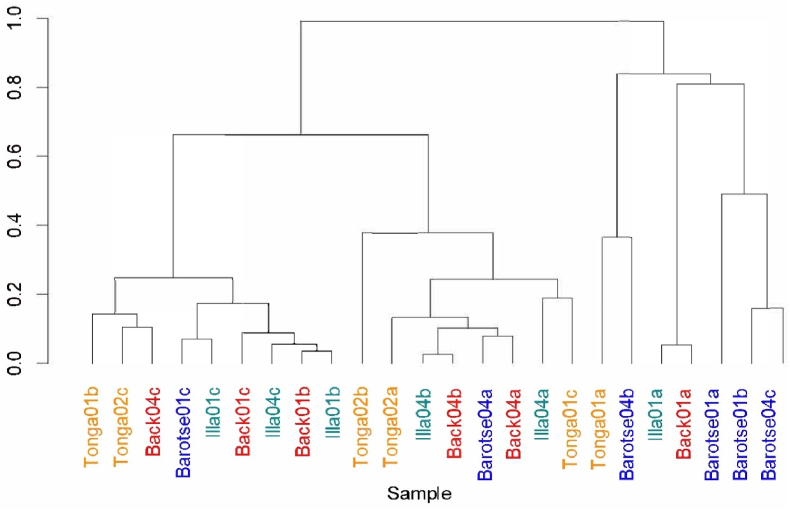
Dendrogram cluster representation of the genus level community dissimilarity for the mabisi variants as measured by Bray-Curtis.

Despite the significant differences between products regarding their volatile compounds, a Bray-Curtis dissimilarity clustering identified minor variations in the microbial communities of the different products (Fig. 7). Barotse samples appeared to be isolated and mostly cluster together, while the other three variants seemed to overlap with each other. Barotse may possess a subtly distinct bacterial community compared to the other variants.

### 3.4 Correlation between odor-active aroma compounds and bacterial genera

**Fig. 8:**
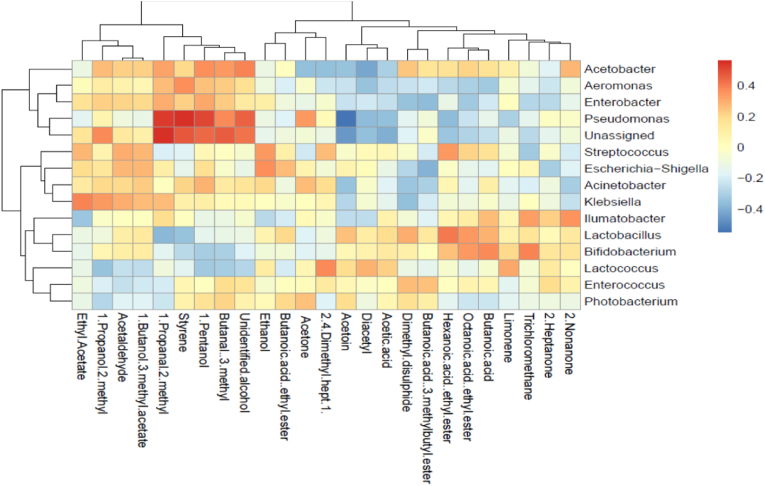
A correlation heatmap and dendrogram matrix displaying the relationship between VOCs (GC-O-MS) and dominant bacterial genera based on the Spearman correlation coefficient. A deep red color indicates a perfect positive correlation, while deep blue indicates a perfect negative correlation.

The potential relationship between the aroma compounds and the bacterial genera in the mabisi samples is illustrated in a heatmap (Fig. 8). Focus was given to the odor-active compounds and correlations were observed between bacterial genera and the volatile compounds. *Acetobacter, Enterobacter, Pseudomonas, Aeromonas,* and the former *Lactobacillus* seemed to correlate with the highest number of odor-active compounds. *Lactococcus* exhibited a limited positive correlation with diacetyl and acetoin. The esters of octanoic acid, hexanoic acid, and butanoic acid show a positive association with *Lactobacillus* and *Streptococcus. Lactobacillus* further correlated positively with butanoic acid, dimethyl-disulphide, and acetoin. Other odor-active volatiles such as 1-propanal-2-methyl, styrene, butanal-3-methyl, and the unidentified alcohol showed a positive correlation with *Pseudomonas* and the unassigned genus. In addition, these volatiles, except styrene, were positively correlated with *Acetobacter*, while styrene was associated with *Aeromonas.*

## 4 Discussion

Our study focused on analyzing the VOCs in four variants of mabisi and identifying key compounds contributing to their characteristic aroma using olfactometry. The study also profiled the microbial community of the four variants using 16S rRNA amplicon sequencing to seek patterns to link microbial composition and functionality. The four mabisi variants are characterized by a common fermented or sour odor (Sikombe et al., 2023). However, their unique volatile compound profiles give each sample a distinct aroma that allows them to be distinguished.

Only twelve of the detected twenty-six volatiles were perceived by olfactometry, highlighting that not all headspace VOCs might contribute to a product's perceived aroma. This is because, despite a response in the MS mode, compound concentrations may be below the odor detection threshold and therefore can not be perceived by the nose. Some VOCs like ethanol and acetaldehyde were undetectable by the nose despite their high concentrations, while others, such as ethyl octanoic acid and 3-methyl-butanal, were odor-active at considerably lower concentrations. Conversely, some compounds that were identified at the sniffing port were not detected in the MS mode; this could probably be due to their low detection thresholds or concentrations below the detection limit of the GC-MS. Similar observations have been reported in other studies (Pang et al., 2012), though potential thermal artefacts during GC analysis could also explain these discrepancies (Baldovini et al., 2020).

Most volatiles identified in our study have also been reported in other fermented dairy products such as kefir and yogurt, as well as traditional fermented products like amasi, and in previous studies on mabisi (Moonga et al., 2021; Benkerroum et al., 2004; Beshkova et al., 2003; Irigoyen et al., 2012; Settachaimongkon et al., 2014; Gadaga et al., 2007). However, 2,4-dimethyl-1-heptene is relatively uncommon in fermented milk products (Cheng, 2010; Jiang et al., 2020). It is a possible contaminant from the fermentation containers as it is reportedly a degradation product of polypropylene (Soják et al., 2007). Dimethyl-disulphide, though uncommon in mabisi, is typical in other fermented dairy products, such as yogurt and cheese, where it contributes a sulphur aroma. In this study, the presence of dimethyl-disulphide was strongly correlated with the genus *Lactobacillus* and *Enterococcus*. However, various authors have attributed its production to the breakdown of methionine by several strains of *Lactococcus* sp. and *Lactobacillus* sp. (Moonga et al., 2021; Ott et al., 1999; Smit et al., 2005; Yvon et al., 2001; Brattoli et al., 2013).

Diacetyl and ethyl octanoic acid were the most common odor-active volatile compounds and were detected in all four variants of mabisi. These compounds contributed buttery, caramel, creamy, and fruity aroma notes to mabisi (Mariaca et al., 2001; West, 2020). Diacetyl is a common volatile compound in fermented dairy products such as yogurt and buttermilk (Tian et al., 2020). Together with acetoin, diacetyl forms the principal metabolic product of the microbial activity on the milk components (Friedrich et al., 1998; Tian et al., 2020; Erkus et al., 2014). These two ketones are associated with certain mesophilic bacteria such as *Lactococcus* sp. and are produced through citrate metabolism (Curioni et al., 2002; McSweeney et al., 2000). These compounds were odor-active in mabisi, which was consistent with the dominance of *Lactococcus* sp. across all the mabisi variants. This highlights the importance of this species in developing the characteristic aroma profiles of mabisi. The dominance of the genus *Lactococcus* in traditional mabisi can be attributed to the production process, which takes place at ambient temperatures of 22–33 °C, thus providing optimum growth conditions for this species (Batt et al., 2014).

The odor-active ethyl esters of butanoic, hexanoic and octanic acids contributed a fruity and sour aroma to mabisi. Fruity notes are desirable aromas in fermented dairy products and they have been associated with ester compounds by many researchers. The fruity notes imparted by the esters can mask undesirable flavors such as bitterness, but they may also be regarded as defects rather than attributes by consumers, although this is concentration and product dependent (Friedrich et al., 1998; Urbach, 1997; Liu et al., 2004; Chen et al., 2017). Fruity notes are defective when the concentration of the ester compounds is high, mostly at ppm levels. From our findings, the concentrations of the ester compounds were notably low, at ppb levels, and it is therefore unlikely that they could be detrimental to the overall product aroma.

A positive correlation was observed between the odor-active ester compounds, and members of the former *Lactobacillus* sp. and *Streptococcus* sp.*,* which is expected from the lipolytic nature of some species of LAB, through the production of enzymes such as lipase and esterase, is attributed to their association with ester formation (McSweeney and Sousa, 2000). In dairy products, lipolysis results from the activity of the indigenous milk lipases or microbial lipase. Esters may be produced from the esterification of fatty acids and alcohols derived from microbial fermentation of lactose or the breakdown of milk fats. However, the role of yeasts in ester production and volatile compound formation, in mabisi, cannot be overlooked. Yeasts are also present in mabisi despite their low abundance (Schoustra et al., 2013). *Lactobacillus* sp*.* was further correlated with butanoic acid, an important flavor component of fermented dairy products and also a common product of the lipolysis of milk fat or the degradation of specific branched-chain amino acids such as leucine, isoleucine, and valine (Curioni et al., 2002; Aiello et al., 2023).

The branched-chain aldehydes, 3-methyl-butanal and 2-methyl-1-propanal, mainly contributed a nutty flavor to mabisi. They have been associated with yogurt aromas and are known as key odorants in some artisanal cheese products (Ayad et al., 1999). Although elevated levels of 3-methyl-butanal above 2 ppm commonly cause off-flavors in some fermented dairy products, in balanced proportion with other volatile compounds, it can enhance the overall aroma profile through synergistic effects (Yvon et al., 2001; Tian et al., 2020; Zhu et al., 2017). These methyl aldehydes are products of the metabolism of the branched-chain amino acids, valine and isoleucine, and are mediated by certain wild strains of *L. lactis* (Ayad et al., 1999).

A comparison of the mabisi variants and their respective cycles showed that barotse4 had a more diverse and intense aroma profile. This observation is in agreement with previous research on the sensory evaluation and consumer acceptance of mabisi, which reported that barotse exhibited a more intense odor compared to the other variants (Sikombe et al., 2023). The observed aroma intensity in barotse4 may be due to its fermentation process, which likely leads to a greater accumulation of metabolites over multiple cycles and enhances the production of key volatile compounds. Such conditions may favor specific microbial groups, driving clear microbial succession—a pattern that is less pronounced in barotse1 and the other variants.

Despite the lack of significant differences in the composition of microbial communities of the samples, the tonga and barotse samples exhibited a trend toward greater community diversity compared to the other variants, aligning with the findings of Moonga and colleagues (Moonga et al., 2020). The lack of distinction is likely due to the genus-level identification being insufficient to reveal these differences; we presume that species-level or lower taxonomical levels, such as genetic lineage, could provide more detailed differences. Unfortunately, this is the limitation of 16S rRNA profiling methods which cannot provide resolution at species and, subsequently, strain level. Moreover, this precise identification was not the main focus of our study as we searched for major general patterns linking microbial composition to function. Additionally, maintaining similar conditions during the production of the different products, including temperature and exposure to the same environmental microbes, may have minimized the differences in community composition. This highlights an alternative approach to using a defined starter culture to drive the microbial community composition by manipulating environmental conditions such as temperature and substrate to achieve specific functional outputs. With this approach, it is possible to investigate the ecological aspects of the mabisi microbiome and its functionality.

Volatile compounds in fermented products are produced via complex metabolic reactions involving enzymatic activities on milk components such as carbohydrates, proteins, and lipids (Smid et al., 2014). A diverse community of lactic acid bacteria drives these metabolic processes and is responsible for the various aromas that are produced. However, the ability of these microorganisms to produce aroma compounds through proteolysis, lipolysis, or citrate metabolism is strain-dependent. The community of microorganisms associated with the volatile compounds in this study may have been a narrow representation of key players in the overall aroma production of mabisi. Other groups of microorganisms, such as yeasts, are also believed to play a role in traditional dairy fermentation and thus aroma formation of these products (Yvon et al., 2001). Identifying odor-active compounds in traditional fermented products and the description of these odors is vital for re-creating these distinct aroma notes, particularly through techniques like the aroma recombination approach (Ott et al., 1999). By understanding the specific odor-active compounds and their contributions to the overall aroma, researchers and producers can accurately replicate the unique and desirable aroma characteristics of different variants of traditional fermented products. This could enhance the optimization of the sensory qualities, thereby helping to preserve the unique flavors of many traditional foods for broader consumer appeal.

The study did not correlate compound concentrations and consumer perception. Establishing the link between compound concentration and aroma perception by consumers is crucial to providing deeper insights into consumer preferences. Therefore, future research on mabisi could focus on this aspect to help pinpoint compound concentrations that produce flavors and off-flavors, and influence consumer perceptions.

## 5 Conclusion

The GC-O-MS and PTR-TOF-MS analyses demonstrated perceivable differences between the four variants of mabisi regarding their odor-active properties and overall volatile profiles, while the composition of the microbial community at the genus level showed minor variations. The study further demonstrates that high detector response or high concentrations do not automatically correspond to compound odor activity as seen by the selectivity of the GC-O-MS towards low odor threshold compounds. This shows that a volatile compound's sensory relevance depends on its headspace concentration and odor detection threshold. Detection in MS mode alone does not confirm odor-activity, but olfactometry identifies volatiles that are key to a product's aroma. This knowledge has far-reaching implications for traditionally fermented products globally. It not only provides valuable insights for tailoring flavor profiles to align with consumer preferences but could also be useful for assessing product quality and detecting incidences of contamination or spoilage in traditional fermented foods.

## CRediT authorship contribution statement

**Thelma W. Sikombe:** Conceptualization, Methodology, Investigation, Data curation, and, Formal analysis, Writing – original draft, Writing – review & editing. **Anita R. Linnemann:** Conceptualization, Resources, Writing – review & editing, Project administration, Funding acquisition, Supervision. **Himoonga B. Moonga:** Writing – review & editing, Project administration, Supervision. **Stefanie Quilitz:** Methodology, Investigation, Data curation, and, Formal analysis. **Sijmen E. Schoustra:** Resources, Writing – review & editing, Project administration, Funding acquisition, Supervision. **Eddy J. Smid:** Resources, Writing – review & editing, Project administration, Funding acquisition, Supervision. **Anna Alekseeva:** Conceptualization, Methodology, Formal analysis, Writing – review & editing.

## Declaration of competing interest

The authors declare that they have no known competing financial interests or personal relationships that could have appeared to influence the work reported in this paper.

## Data Availability

Data will be made available on request.
