# Supplementary material for: Odor-active aroma compounds in traditional fermented dairy products: The case of mabisi in supporting food and nutrition security in Zambia
Source: Curr Res Food Sci. 2025 Jan 16;10:100976. doi: 10.1016/j.crfs.2025.100976 (PMC11795106; doi:10.1016/j.crfs.2025.100976)
Supplement: Multimedia component 1 [file mmc1.pdf]

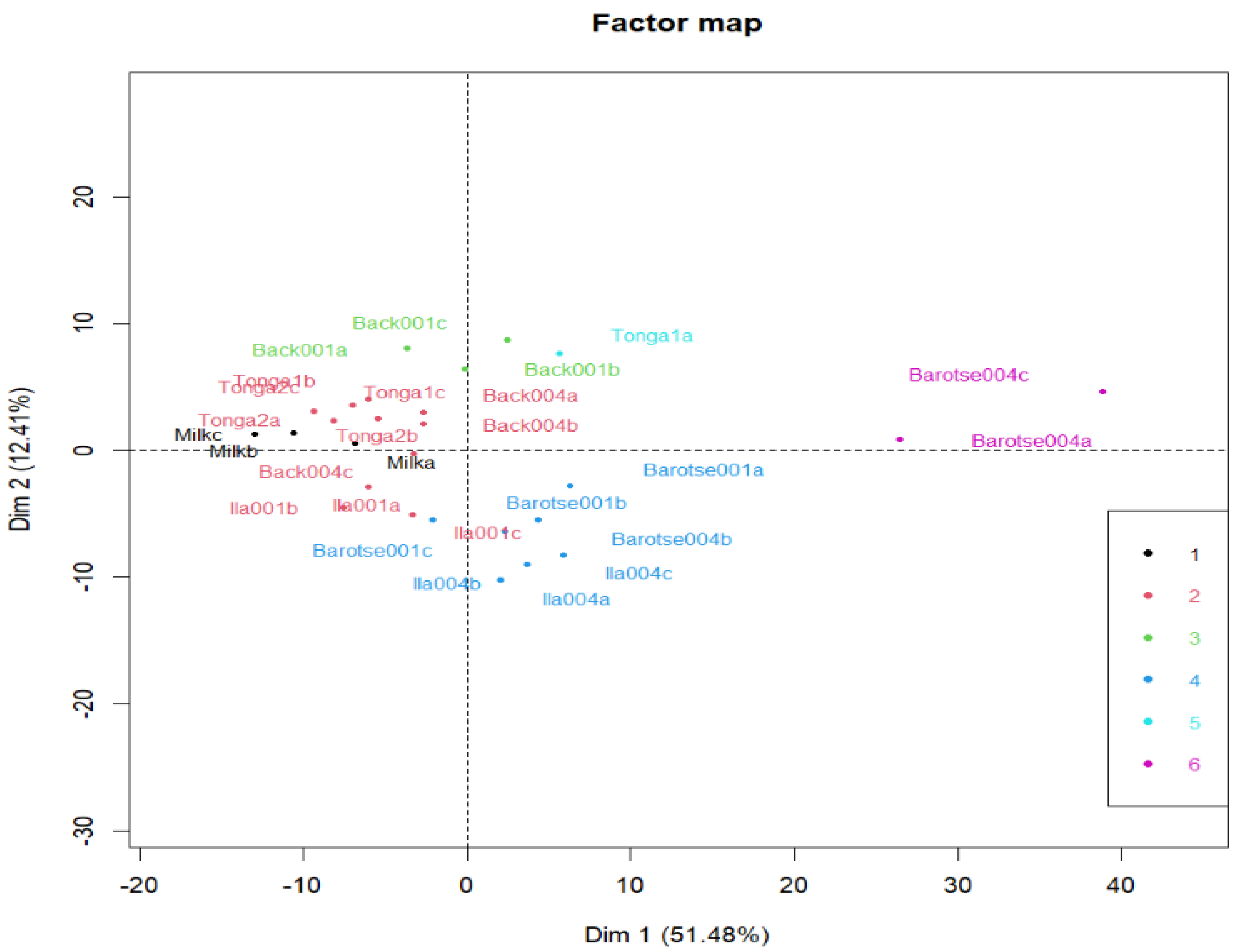

Fig. S1. Clusters of the mabisi variants and their respective production cycles based on normalized data (concentrations of VOC (ppbv)) obtained by PTR-QiTOF-MS measurements. The clustering was performed with HCPC function in R on PCA results. Color variations represent the six different clusters that were formed.
