# Supplementary material for: Odor-active aroma compounds in traditional fermented dairy products: The case of mabisi in supporting food and nutrition security in Zambia
Source: Curr Res Food Sci. 2025 Jan 16;10:100976. doi: 10.1016/j.crfs.2025.100976 (PMC11795106; doi:10.1016/j.crfs.2025.100976)
Supplement: Multimedia component 2 [file mmc2.pdf]

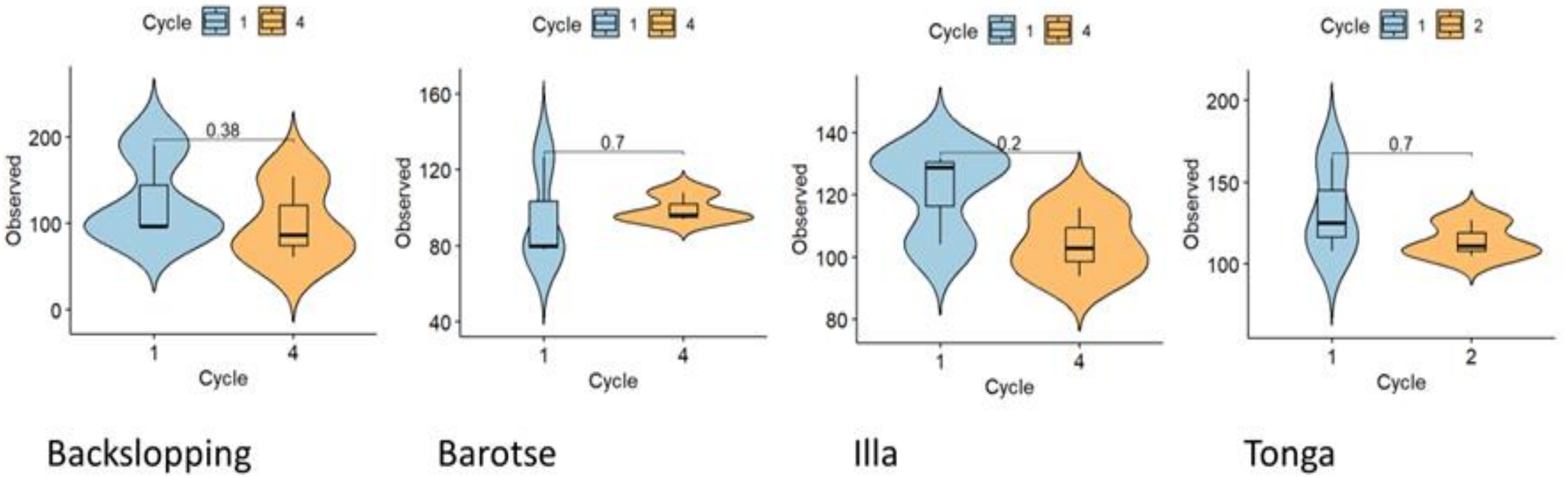

Fig. S2. Observed Alpha diversity, showing the variation in the species richness of bacterial community between cycles of the mabisi products. $p > 0.05$
