# Supplementary material for: Odor-active aroma compounds in traditional fermented dairy products: The case of mabisi in supporting food and nutrition security in Zambia
Source: Curr Res Food Sci. 2025 Jan 16;10:100976. doi: 10.1016/j.crfs.2025.100976 (PMC11795106; doi:10.1016/j.crfs.2025.100976)
Supplement: Multimedia component 3 [file mmc3.docx]

Table S1. Means and standard deviations of peak area percentages of volatiles identified by GC-MS during GC-O-MS measurements in the different types of mabisi. Relative peak areas were obtained by calculating a percentage of each peak of the total area of all peaks in a sample. Significant differences between samples are indicated by different letters (Kruskal-Wallis rank sum test, Bonferroni, α=0.05). Additionally, the linear retention index (LRI) of the compounds on a Stabilwax® DA column are given.

| Compound | LRI | Peak area percentages (Mean ± SD) | | | | | | | | Identification |
| --- | --- | --- | --- | --- | --- | --- | --- | --- | --- | --- |
|  |  | Illa1 | Illa4 | Backlopping1 | Backlopping4 | Barotse1 | Barotse4 | Tonga1 | Tonga2 |  |
| Acetaldehyde** | 703.44 | 0.00 | 0.00 | 0.00 | 0.00 | 0.00 | 0.90 ± 0.55^a^ | 0.00 | 0.00 | LRI, MS |
| 1-Propanal-2-methyl** | 823.40 | 0.00 | 0.00 | 0.00 | 0.00 | 0.00 | 0.00 | 1.53 ± 1.3^a^ | 2.93 ± 0.7^a^ | LRI, MS, GCO |
| Acetone^ns^ | 826.43 | 4.57 ± 1.9 | 4.26 ± 1.4 | 3.49 ± 0.3 | 3.59 ± 1.0 | 1.85 ± 0.8 | 0.00 | 4.52 ± 1.5 | 3.32 ± 0.4 | LRI, MS |
| 2,4-Dimethyl-hept-1-ene* | 884.84 | 13.3 ± 10.7^a^ | 7.81 ± 1.7^a^ | 1.64 ± 2.8^ab^ | 2.59 ± 2.4^ab^ | 1.91 ± 0.6^ab^ | 0.57 ±0.6^ab^ | 2.82 ±1.0^ab^ | 1.84 ± 1.6^ab^ | LRI, MS |
| Ethyl-Acetate* | 898.99 | 0.50 ± 0.9^ab^ | 0.00 | 0.00 | 1.19 ± 1.2^ab^ | 0.00 | 7.95 ± 0.8^a^ | 0.59 ±1.0^ab^ | 0.00 | LRI, MS |
| Butanal-3-methyl** | 932.16 | 0.00 | 0.00 | 0.00 | 0.57 ± 1.0^cd^ | 3.6 ± 0.7^ab^ | 1.06 ±0.2^bc^ | 28.46 ±3.1^a^ | 25.8 ± 1.7^a^ | LRI, MS,GCO |
| Ethanol** | 941.81 | 18.26 ± 15^ab^ | 5.94 ± 0.2^de^ | 16.55 ± 4.2^ab^ | 19.28 ± 2.7^a^ | 5.8 ± 0.8^de^ | 43.9 ± 2.3^a^ | 6.75 ± 0.3^cd^ | 7.13 ± 1.0^bc^ | LRI, MS |
| Diacetyl^ns^ | 993.65 | 2.05 ± 1.8 | 2.93 ± 1.5 | 2.30± 0.8 | 2.33± 0.6 | 0.85± 0.8 | 0.26± 0.2 | 1.10± 1.0 | 0.00 | LRI, MS, GCO |
| Trichloromethane^ns^ | 1037.62 | 12.7 ± 14 | 15.92 ±12.2 | 13.78 ± 8.4 | 8.21 ± 6.2 | 5.58 ± 9.7 | 0.15 ± 0.2 | 1.55 ± 1.3 | 10.60 ± 9.68 | LRI, MS |
| Butanoic acid-ethy ester* | 1053.55 | 1.67 ± 0.34^abc^ | 1.78 ±1.87^ab^ | 2.97 ± 1.53^abc^ | 3.6 ± 1.46^ab^ | 5.12 ± 0.74^a^ | 2.86 ±0.49^ab^ | 1.64±0.36^abc^ | 1.33 ± 0.63^bc^ | LRI, MS, GCO |
| 1-Propanol-2-methyl** | 1103.07 | 0.00 | 0.00 | 0.32 ± 0.56^ab^ | 0.00 | 0.00 | 2.2 ± 0.26^a^ | 1.31 ±1.13^ab^ | 0.24 ± 0.41^ab^ | LRI, MS |
| Dimethyl-disulphide** | 1104.82 | 0.00 | 0.00 | 0.00 | 0.00 | 2.92 ± 0.35^a^ | 0.00 | 0.00 | 0.00 | LRI, MS,GCO |
| 1-Butanol-3-methyl acetate ** | 1141.05 | 0.00 | 0.00 | 0.00 | 0.00 | 0.00 | 0.56 ± 0.23^a^ | 0.00 | 0.00 | LRI, MS |
| 2-Heptanone** | 1202.68 | 6.09 ± 2.39^ab^ | 7.7 ± 2.67^a^ | 0.66 ± 1.15^cd^ | 0.84 ± 0.75^cd^ | 3.30 ± 0.30^ab^ | 0.00 | 1.78±0.35^bcd^ | 1.22 ± 1.13^cd^ | LRI, MS |
| 1-Pentanol** | 1213.462 | 2.86 ± 2.55^de^ | 3.36 ±1.69^de^ | 8.28 ± 4.04^cd^ | 10.8 ± 4.46^bc^ | 13.3 ± 2.34^bc^ | 17.6±0.14^ab^ | 19.8±1.62^a^ | 20.1 ± 1.62^a^ | LRI, MS |
| Limonene^ns^ | 1224.10 | 4.98 ± 5.96 | 15.5± 26.8 | 4.77 ± 5.02 | 0.62 ± 1.07 | 0.32 ± 0.56 | 0.86 ± 0.25 | 0.73 ± 1.26 | 0.81 ± 1.41 | LRI, MS |
| Hexanoic acid-ethyl ester** | 1248.80 | 7.68 ± 1.73^abc^ | 5.97±0.62^bcd^ | 4.41 ± 0.81^de^ | 4.59 ± 0.72^cd^ | 9.05 ± 0.53^ab^ | 10.03 ±0.48^a^ | 2.54 ± 0.94^ef^ | 2.12 ± 0.15^ef^ | LRI, MS, GCO |
| Butanoic acid-3-methylbutyl ester** | 1281.09 | 0.00 | 0.00 | 0.00 | 0.00 | 2.02 ± 0.28^a^ | 0.00 | 0.28 ± 0.48^b^ | 0.00 | LRI, MS |
| Styrene** | 1288.31 | 0.00 | 0.00 | 0.00 | 0.00 | 0.00 | 0.00 | 1.71 ± 0.23^a^ | 1.33 ± 0.49^a^ | LRI, MS, GCO |
| Acetoin** | 1313.94 | 1.45 ± 0.48^c^ | 2.55±1.25^bc^ | 29.9 ± 2.55^a^ | 30.6 ± 3.9^a^ | 8.33 ± 1.58^ab^ | 1.65 ± 0.08^c^ | 0.00 | 0.00 | LRI, MS, GCO |
| 2-Nonanone^ns^ | 1313.87 | 0.47 ± 0.81 | 0.95 ± 1.65 | 0.48 ± 0.84 | 0.00 | 0.97 ± 0.12 | 0.00 | 0.00 | 0.34 ± 0.59 | LRI, MS |
| Octanoic acid-ethyl ester** | 1450.82 | 2.76 ± 0.45^ab^ | 1.23 ±1.17^bc^ | 0.11 ± 0.2^c^ | 0.86 ± 0.76^bc^ | 9.87 ± 1.01^a^ | 2.75 ±0.35^ab^ | 0.00 | 0.23 ± 0.39^c^ | LRI, MS, GCO |
| Acetic acid** | 1469.55 | 11.5 ± 4.26^ab^ | 18.9 ± 4.70^a^ | 10.36 ± 2.04^bc^ | 10.27 ± 1.23^bc^ | 13.41 ± 1.42^ab^ | 5.07 ±0.47^cd^ | 2.78 ±0.33^de^ | 1.53 ± 1.39^de^ | LRI, MS |
| Unidentified alcohol** | 1505.55 | 0.00 | 0.00 | 0.00 | 0.00 | 7.28 ± 0.6^b^ | 1.28 ± 0.12^b^ | 19.7 ± 0.57^a^ | 19.1 ± 1.98^a^ | LRI, MS, GCO |
| Butanoic acid** | 1645.01 | 9.12 ± 7.21^a^ | 5.22 ±1.56^ab^ | 0.00 | 0.00 | 4.49 ± 1.05^ab^ | 0.34 ± 0.3^bc^ | 0.43 ± 0.74^c^ | 0.00 | LRI, MS, GCO |

*^ns^ p-value > 0.05 *p-value ≤ 0.05 **p-value ≤ 0.01 ***p-value ≤ 0.001*
