# Supplementary material for: Odor-active aroma compounds in traditional fermented dairy products: The case of mabisi in supporting food and nutrition security in Zambia
Source: Curr Res Food Sci. 2025 Jan 16;10:100976. doi: 10.1016/j.crfs.2025.100976 (PMC11795106; doi:10.1016/j.crfs.2025.100976)
Supplement: Multimedia component 4 [file mmc4.docx]

Table S2. Tentatively identified m/z peaks, measured by PTR-QiTOF-MS, that differentiated the mabisi products significantly and were significant in describing specific samples.

| Measured m/z | Theoret. m/z | Sum  formula | Tentative Identification | Chemical class | Conc. (ppb) Mean ± SD | |
| --- | --- | --- | --- | --- | --- | --- |
| **Backslopping001** | | | | | | |
| 33.0335*** | 33.033 | CH_4_OH^+^ | Methanol^1^# | Alcohols | 61.48 ± 3.57 |  |
| 69.0335*** | 69.034 | C_4_H_4_OH^+^ | Furan^1^ | Furan | 0.22 ± 0.02 |  |
| 71.0495*** | 71.049 | C_4_H_6_OH^+^ | Butenal^1^ | Aldehydes | 135.05 ± 22.92 |  |
| 87.044*** | 87.0441 | C_4_H_6_O_2_H^+^ | Diacetyl^1-3^ | Ketone | 8.67 ± 1.19 |  |
| 89.0602*** | 89.0604 | C_4_H_8_O_2_H^+^ | Acetoin/Ethyl acetate/Butanoic acid^2^ | Ketones/Esters/Acids | 151.47 ± 24.32 |  |
| 91.0592* | 91.058 | C_4_H_10_SH^+^ | Diethylsulphide/Butanethiol (fragment)^1, 2^ | Sulphur compounds | 1.34 ± 0.27 |  |
| 93.0695* | 93.037 | C_7_H_8_H^+^ | Toluene^1, 2^ | Aromatic hydrocarbons | 1.02 ± 0.09 |  |
| **Backslopping004** | | | | | | |
| 71.0495*** | 71.049 | C_4_H_6_OH^+^ | Butenal^1^ | Aldehydes | 59.77 ± 16.46 |  |
| 89.0602** | 89.0604 | C_4_H_8_O_2_H^+^ | Acetoin/Ethyl acetate/Butanoic acid^2^ | Ketones/Esters/Acids | 69.33 ± 17.58 |  |
| **Barotse001** | | | | | | |
| 60.0216*** | 60.0212 | C_2_H_3_O_2_H^+^ | Acetate^4^ | Esters | 0.84 ± 0.22 |  |
| 81.0697** | 81.070 | C_6_H_8_H^+^ | Cyclohexadiene^1^ | Terpene fragment | 1.45 ± 0.05 |  |
| 95.0148*** | 95.016 | C_2_H_6_O_2_SH^+^ | Dimethyl sulfone (Methylsulfonylmethane)^1^ | Sulphur compouds | 0.91 ± 0.49 |  |
| 99.0806*** | 99.080 | C_6_H_10_OH^+^ | Hexenal/Methylpentenone^1^ | Aldehydes/Ketones | 1.01 ± 0.12 |  |
| 109.1011** | 109.101 | C_8_H_12_H^+^ | Cyclooctadiene^1^ | Hydrocarbons | 1.96 ± 0.18 |  |
| 110.1045** | 111.044 | C_6_H_6_O_2_H^+^ | Acetyl furan^1^ | Furans | 0.18 ± 0.02 |  |
| 117.0911*** | 117.0917/ 117.0913 | C_6_H_12_O_2_H^+^ | Ethyl butanoic acid/Hexanoic acid^2^ | Esters/Acids | 4.53 ± 0.74 |  |
| 123.0461* | 123.0811 | C_8_H_10_OH^+^ | Phenylethyl Alcohol | Alcohols | 0.42 ± 0.04 |  |
| 127.1119*** | 127.1488 | C_9_H_18_H^+^ | 2,4-Dimethylhept-1-ene | Alkenes | 1.21 ± 0.08 |  |
| 137.1331* | 137.133 | C_10_H_16_H^+^ | Various monoterpenes^1^ | Terpenes | 1.01 ± 0.03 |  |
| 201.1764** | 201.1856 | C_12_H_24_O_2_H^+^ | Decanoic acid, ethyl ester | Esters | 0.05 ± 0.01 |  |
| **Barotse004** | | | | | | |
| 43.054*** |  |  | Alkyl fragment^2^ |  | 114.99 ± 76.37 |  |
| 44.0209*** |  |  | Acetic acid fragment^5^ | Acid fragment | 16.68 ± 8.93 |  |
| 45.0336*** | 45.034 | C_2_H_4_OH^+^ | Acetaldehyde^1^ | Aldehydes | 1096.83 ± 815.01 |  |
| 47.0492*** | 47.049 | C_2_H_5_OH^+^ | Ethanol^1^ | Alcohols | 2331.85 ± 1853.20 |  |
| 57.0699*** |  |  | Pentanoic acid fragment^5^ | Acid fragment | 71.98 ± 51.42 |  |
| 60.0216*** | 60.0212 | C_2_H_3_O_2_H^+^ | Acetate^4^ | Esters | 0.86 ± 0.32 |  |
| 61.0287*** | 61.028 | C_2_H_4_O_2_H^+^ | Acetic acid^1^ | Acids | 638.84 ± 307.61 |  |
| 69.0335* | 69.034 | C_4_H_4_OH^+^ | Furan^1^ | Furan | 0.21 ± 0.02 |  |
| 71.0855*** |  |  | Butanoic acid fragment/Pentanoic acid fragment^5^ | Acid fragments | 37.05 ± 28.35 |  |
| 73.0291* | 73.0290 | C_3_H_4_O_2_H^+^ | 2-Propenoic acid^4^ | Acids | 0.87 ± 0.20 |  |
| 73.0646*** | 73.065 | C_4_H_8_OH^+^ | Methylpropanal/2-Butanone/Butanal^1, 2^ | Aldehydes/Ketones | 28.94 ± 16.02 |  |
| 75.0437*** | 75.044 | C_3_H_6_O_2_H^+^ | Propionic acid^1, 5^ | Acids | 2.70 ± 0.83 |  |
| 75.0803*** | 75.0811 | C_4_H_10_OH^+^ | 1-Propanol,2-methyl | Alcohols | 3.18 ± 1.90 |  |
| 81.0697*** | 81.070 | C_6_H_8_H^+^ | Cyclohexadiene^1^ | Terpene fragment | 1.58 ± 0.11 |  |
| 85.0647** | 85.065 | C_5_H_8_OH^+^ | Pentenal/Pentenone^1^ | Aldehydes/Ketones | 0.91 ± 0.46 |  |
| 85.1008* | 85.1018 | C_6_H_12_H^+^ | Cyclohexane^4^ | Alkanes | 0.50 ± 0.12 |  |
| 87.044** | 87.0441 | C_4_H_6_O_2_H^+^ | Diacetyl^1-3^ | Ketone | 6.69 ± 4.14 |  |
| 91.0592*** | 91.058 | C_4_H_10_SH^+^ | Diethylsulphide/Butanethiol (fragment)^1^ | Sulphur compounds | 1.68 ± 0.92 |  |
| 93.0366*** | 93.037 | C_7_H_8_H^+^ | Toluene^1, 2^ | Aromatic hydrocarbons | 2.08 ± 0.30 |  |
| 97.0286** | 97.028 | C_5_H_4_O2H^+^ | Furfural^1^ | Aldehydes | 0.22 ± 0.02 |  |
| 97.0639*** | 97.065 | C_6_H_8_OH^+^ | Hexadienal/Ethylfuran^1, 2^ | Aldehydes/Furans | 0.38 ± 0.15 |  |
| 97.101* |  |  | Heptanal fragment |  | 1.08 ± 0.35 |  |
| 99.0806*** | 99.080 | C_6_H_10_OH^+^ | Hexenal/Methylpentenone/Hexanoic acid fragment^1, 5^ | Aldehydes/Ketones/Acid fragment | 1.14 ± 0.39 |  |
| 101.0601*** | 101.0603 | C_5_H_8_O_2_H^+^ | Pentenoic acid^4^ | Acids | 2.60 ± 1.12 |  |
| 103.0401* | 103.0396 | C_4_H_6_O_3_H^+^ | Acetyl acetate^4^ | Esters | 0.14 ± 0.02 |  |
| 103.0755*** | 103.075 | C_5_H_10_O_2_H^+^ | Methylbutanoic acid/Pentanoic acid^1, 5^ | Acids | 1.05 ± 0.39 |  |
| 105.0357* | 105.037 | C_4_H_8_OSH^+^ | Methional^1^ | Sulphur compounds | 0.21 ± 0.06 |  |
| 105.0708*** | 105.070 | C_8_H_8_H^+^ | Styrene/Ethylbenzene/Vinylbenzene^1^ | Aromatic hydrocarbons | 1.50 ± 0.71 |  |
| 109.066** | 109.065 | C_7_H_8_OH^+^ | Benzyl alcohol (cresol)^1^ | Phenols | 0.17 ± 0.05 |  |
| 109.1011*** | 109.101 | C_8_H_12_H^+^ | Cyclooctadiene^1^ | Hydrocarbons | 2.01 ± 0.33 |  |
| 110.1045*** | 111.044 | C_6_H_6_O_2_H^+^ | Acetyl furan^1^ | Furans | 0.19 ± 0.03 |  |
| 117.0911*** | 117.0917/ 117.0913 | C_6_H_12_O_2_H^+^ | Ethyl butanoic acid/Hexanoic acid^2, 5^ | Esters/Acids | 5.23 ± 1.98 |  |
| 123.0461** | 123.0811 | C_8_H_10_OH^+^ | Phenylethyl Alcohol | Alcohols | 0.44 ± 0.13 |  |
| 125.0947*** | 125.0954 | C_6_H_10_N_3_H^+^ | 2-Methoxyphenol (Guaiacol)^4^ | Phenols | 0.20 ± 0.03 |  |
| 127.1119*** | 127.1488 | C_9_H_18_H^+^ | 2,4-Dimethylhept-1-ene | Alkenes | 1.25 ± 0.08 |  |
| 136.1061*** | 131.107 | C_7_H_14_O_2_H^+^ | Heptanoic acid/Hexyl formate^1^ | Acids/Esters | 0.16 ± 0.05 |  |
| 137.1331*** | 137.133 | C_10_H_16_H^+^ | Various monoterpenes^1^ | Terpenes | 1.10 ± 0.08 |  |
| 143.144* | 143.143 | C_9_H_18_OH^+^ | Nonanone/Nonanal^1, 2, 5^ | Ketones/Aldehydes | 0.79 ± 0.17 |  |
| 145.1232*** | 145.1230 | C_8_H_16_O_2_H^+^ | Ethyl hexanoic acid/Octanoic acid | Esters/Acids | 2.27 ± 1.57 |  |
| 153.127*** | 153.127 | C_10_H_16_OH^+^ | Decadienal^1^ | Aldehydes | 0.22 ± 0.02 |  |
| 165.091*** | 165.0917 | C_10_H_12_O_2_H^+^ | Acetic acid, 2-phenylethyl ester | Esters | 0.04 ± 0.00 |  |
| 166.0876* | 166.0869 | C_9_H_11_NO_2_H^+^ | Phenylalanine^4^ | Amino Acids | 0.01 ± 0.00 |  |
| 173.1521*** | 173.1541 | C_10_H_20_O_2_H^+^ | Ethyl octanoic acid/n-decanoic acid^5^ | Esters/Acids | 0.51 ± 0.34 |  |
| 201.1764*** | 201.1856 | C_12_H_24_O_2_H^+^ | Decanoic acid, ethyl ester | Esters | 0.08 ± 0.03 |  |
| **Illa001** | | | | | | |
| 109.1011*** | 109.101 | C_8_H_12_H^+^ | Cyclooctadiene^1^ | Hydrocarbons | 2.05 ± 0.12 |  |
| 110.1045*** | 111.044 | C_6_H_6_O_2_H^+^ | Acetyl furan^1^ | Furans | 0.19 ± 0.01 |  |
| 127.1119*** | 127.1488 | C_9_H_18_H^+^ | 2,4-Dimethylhept-1-ene | Alkenes | 1.12 ± 0.05 |  |
| **Illa004** | | | | | | |
| 60.0216* | 60.0212 | C_2_H_3_O_2_H^+^ | Acetate^4^ | Esters | 0.73 ± 0.17 |  |
| 81.0697** | 81.070 | C_6_H_8_H^+^ | Cyclohexadiene^1^ | Terpene fragment | 1.49 ± 0.06 |  |
| 99.0806*** | 99.080 | C_6_H_10_OH^+^ | Hexenal/methylpentenone^1^ | Aldehydes | 1.10 ± 0.11 |  |
| 109.1011*** | 109.101 | C_8_H_12_H^+^ | Cyclooctadiene^1^ | Hydrocarbons | 2.05 ± 0.33 |  |
| 117.0911*** | 117.0917/ 117.0913 | C_6_H_12_O_2_H^+^ | Ethyl butanoic acid/ Hexanoic acid^2^ | Esters/Acids | 5.09 ± 0.49 |  |
| 123.0461** | 123.0811 | C_8_H_10_OH^+^ | Phenylethyl Alcohol | Alcohols | 0.43 ± 0.04 |  |
| 127.1119*** | 127.1488 | C_9_H_18_H^+^ | 2,4-Dimethylhept-1-ene | Alkenes | 1.23 ± 0.12 |  |
| 136.1061*** | 131.107 | C_7_H_14_O_2_H^+^ | Heptanoic acid/Hexyl formate^1^ | Acids/Esters | 0.16 ± 0.02 |  |
| 153.127** | 153.127 | C_10_H_16_OH^+^ | Decadienal^1^ | Aldehydes | 0.19 ± 0.00 |  |
| **Tonga1** | | | | | | |
| 69.0699** | 69.070 | C_5_H_8_H^+^ | Isoprene^1, 2^ | Terpene fragment | 23.85 ± 24.75 |  |
| 85.1008* | 85.1018 | C_6_H_12_H^+^ | Cyclohexane^4^ | Alkanes | 0.49 ± 0.19 |  |
| 87.0801** | 87.0811 | C_5_H_10_OH^+^ | 3-methyl butanal/2-Pentanone/Pentanal^2^ | Aldehydes/Ketones/Aldehydes | 5.05 ± 5.45 |  |
| 93.0695** | 93.070 | C_7_H_8_H^+^ | Toluene^1, 2^ | Aromatic hydrocarbons | 1.06 ± 0.09 |  |
| 95.0855*** | 95.086 | C_7_H_10_H^+^ | Methylcyclohexadiene (α-terpinene fragment)^1^ | Terpenes | 1.22 ± 0.61 |  |
| 103.0755* | 103.075 | C_5_H_10_O_2_H^+^ | Methylbutanoic acid/Pentanoic acid^1, 5^ | Acids | 0.88 ± 0.26 |  |
| 111.0796*** | 11.080 | C_7_H_10_OH^+^ | Heptadienal^1^ | Aldehydes | 0.37 ± 0.07 |  |
| 113.0962*** | 113.096 | C_7_H_12_OH^+^ | Heptenal^1^ | Aldehydes | 3.02 ± 0.03 |  |
| 121.0656** | 121.065 | C_8_H_8_OH^+^ | Methylbenzaldehydecoumaran^1^ | Aldehydes | 0.85 ± 0.44 |  |
| 131.1067*** | 131.107 | C_7_H_14_O_2_H^+^ | Heptanoic acid/Hexyl formate^1^ | Acids/Esters | 2.77 ± 1.24 |  |
| **Tonga2** | | | | | | |
| 93.0695** | 93.070 | C_7_H_8_H^+^ | Toluene^1, 2^ | Aromatic hydrocarbons | 1.04 ± 0.07 |  |
| 95.0855** | 95.086 | C_7_H_10_H^+^ | Methylcyclohexadiene (α-terpinene fragment)^1^ | Terpenes | 0.96 ± 0.13 |  |
| 111.0796** | 11.080 | C_7_H_10_OH^+^ | Heptadienal | Aldehydes | 0.27 ± 0.05 |  |
| 113.0962** | 113.096 | C_7_H_12_OH^+^ | Heptenal^1^ | Aldehydes | 2.07 ± 0.39 |  |
| 131.1067*** | 131.107 | C_7_H_14_O_2_H^+^ | Heptanoic acid/Hexyl formate^1^ | Acids/Esters | 2.15 ± 0.39 |  |

**Significance code: *p-value ≤ 0.05 **p-value ≤ 0.01 **p-value ≤ 0.001*

*^#^ denotes references*

Of the 390 mass peaks obtained, 218 were distinct for barotse4, and 35 mass peaks were distinct for barotse1. Backslopping1 and 4 had 31 and 9 mass peaks respectively, while only 5 mass peaks were distinct for illa1. Illa4 had 67 mass peaks. Tonga1 and tonga2 were described by 36 and 16 mass peaks, respectively. More of the tentatively identified compounds, including those with mass peaks at m/z 123.0461, m/z 165.0910, m/z 145.1232, m/z 117.0911, and m/z 201.1764, and identified tentatively as phenylethyl alcohol, acetic acid 2-phenylethyl ester, and decanoic acid ethyl ester significantly distinguished barotse4 from the other mabisi products in the PTR-QiTOF-MS analysis.

References

1. Yener S, Sánchez-López JA, Granitto PM, Cappellin L, Märk TD, Zimmermann R, et al. Rapid and direct volatile compound profiling of black and green teas (Camellia sinensis) from different countries with PTR-ToF-MS. Talanta. 2016;152:45-53.

2. Bottiroli R, Pedrotti M, Aprea E, Biasioli F, Fogliano V, Gasperi F. Application of PTR-TOF-MS for the quality assessment of lactose-free milk: Effect of storage time and employment of different lactase preparations. J Mass Spectrom. 2020;55(11):e4505.

3. Soukoulis C, Aprea E, Biasioli F, Cappellin L, Schuhfried E, Märk TD, et al. Proton transfer reaction time-of-flight mass spectrometry monitoring of the evolution of volatile compounds during lactic acid fermentation of milk. Rapid Commun Mass Spectrom. 2010;24(14):2127-3134.

4. Patiny L, Borel A. ChemCalc: A Building Block for Tomorrow’s Chemical Infrastructure. Journal of Chemical Information and Modeling. 2013;53(5):1223-8.

5. Aprea E, Romano A, Betta E, Biasioli F, Cappellin L, Fanti M, et al. Volatile compound changes during shelf life of dried Boletus edulis: comparison between SPME-GC-MS and PTR-ToF-MS analysis. J Mass Spectrom. 2015;50(1):56-64.
